# Torsion of gall bladder, a rare entity: a case report and review article

**DOI:** 10.1186/1757-1626-2-193

**Published:** 2009-11-12

**Authors:** Vanita Gupta, Vikrant Singh, Ajit Sewkani, Dipak Purohit, Rajneesh Varshney, Subodh Varshney

**Affiliations:** 1 Department of Anatomy, Peoples College of Medical Sciences and Research Centre, Bhopal, Madhya Pradesh, India; 2 Department of Surgical Gastroenterology & Clinical Nutrition, Bhopal Memorial Hospital and Research Centre, Bhopal, Madhya Pradesh, India

## Abstract

**Introduction:**

Gallbladder torsion is a rare entity, which is often difficult to diagnose preoperatively. Since its first description in 1898 by Wendel, there have been over 500 documented cases in the literature. It is defined as rotation of the gallbladder on its mesentery along the axis of the cystic duct and cystic artery. Gallbladder torsion is more frequently encountered in the elderly with peak incidence in the 65-75 year old group, and a 3:1 female predominance. Gallbladder torsion typically presents as an acute abdomen requiring emergency surgery, and most cases are found as a surprise at surgery since preoperative diagnosis of gallbladder torsion is difficult. We report a case of acute gallbladder torsion in an elderly male and review the clinical aspect of gallbladder torsion.

**Case report:**

A 54-year old male presented to our department with a 5-day history of sudden onset colicky abdominal pain associated with vomiting, progressive abdominal distension and fever. Laparotomy through a chevron incision was performed and findings at operation included a gallbladder, which was necrotic and gangrenous, not attached to the liver by any mesentery. It was hanging by the attachments of cystic duct and cystic artery only, with a 360-degree clockwise torsion.

**Conclusion:**

Gallbladder torsion is rare surgical emergency which requires a high index of suspicion for early preoperative diagnosis and prompt intervention. Treatment consists of cholecystectomy with a prior detorsion to avoid injury to the common duct.

## Introduction

Gallbladder torsion is defined as the rotation of the gallbladder on its mesentery along the axis of the cystic duct and cystic artery [1]. It is an uncommon clinical entity and a difficult condition to diagnose preoperatively. Since its first description in 1898 by Wendell, there have been over 500 documented cases in the literature. The case reported by Wendell was a floating gallbladder, with a long cystic duct, and a floating kidney, with cholelithiasis and perforation of the gall-bladder [2]. Etiologically, two types of gallbladder have a tendency to undergo volvulus - those with a wide mesentery and those in which the mesentery covers only the cystic duct and artery. Both of these conditions allow the gallbladder to float and result in volvulus. Loss of fat and the liver atrophy that may occur with advancing age can cause an acquired gallbladder mesentery [3]. The clinical features can be grouped into three triads: a triad of the patient's characteristics which consists of a thin, old patient with chronic chest disease or a deformed spine; a triad of symptoms which consists of typical abdominal pain, early onset of vomiting and a short history; and a triad of physical signs which consists of an abdominal mass, a lack of toxaemia or jaundice and a discrepancy in the pulse and temperature [4]. Imaging studies may contribute to the diagnosis but are often nonspecific. Ultrasound scan will normally identify the enlarged gallbladder inferior to its normal anatomical position with a thickened wall and surrounded by free fluid. Magnetic resonance (MR) imaging findings include high signal intensity within the gallbladder wall on T1-weighted images suggesting haemorrhagic infarct and necrosis [5].

## Case presentation

A 54-year old male presented to our department with a 5-day history of sudden onset colicky abdominal pain associated with vomiting, progressive abdominal distension and fever. He had failed to pass any faeces or flatus 24 hours prior to presentation. He denied of any history of recent change in bowel habit or weight loss. There was no significant relevant past medical history. He was admitted with stable vital signs. Abdominal examination revealed a palpable tender right hypochondrial mass measuring 5 cm by 6 cm. Bowel sounds and digital rectal examinations were normal. Laboratory blood tests revealed mildly deranged liver function tests [Bilirubin 25 μmol/L, ALP 276 U/L, ALT 277 U/L & GGT 301 U/L], and white blood cell count of 20 × 10^9 ^(neutrophils 17.8 × 10^9^). Computerized tomography (CT) scan of the abdomen and pelvis revealed mass at neck of gall bladder. He was admitted with the provisional diagnosis of carcinoma gall bladder.

**Figure 1:**
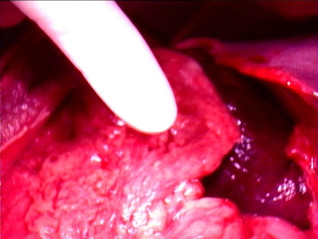
**torsion of gallbladder**.

The decision was then taken to proceed with Radical cholecystectomy. Laparotomy through a chevron incision was performed and findings at operation included a gallbladder, which was gangrenous not attached to the liver (figure 1). It was hanging by the attachments of cystic duct and cystic artery only, with a 360-degree clockwise torsion. The gallbladder was distinctly suspended free from the liver edge and lying over the greater omentum and transverse colon. The pedicle was derotated and cholecystectomy was performed. (Additional file 1) The opened gallbladder had wall thickness of about 8 mm. The pathological examination of the specimen revealed a 10 cm enlarged gallbladder; 5.6 cm in diameter, with a 1 cm thick edematous wall with mucosal congestion and the histological findings were consistent with acute gangrenous cholecystitis with extensive necrosis of the wall. Patient had drain of 300 ml on first postoperative day, 100 ml on second postoperative day and 20 ml on third postoperative day. Patient was discharged on third postoperative day after removal of drain. Patient had an uneventful postoperative course.

## Discussion

A volvulus is the twisting of a nonsolid organ around its mesenteric axis. Volvulus most commonly occurs at the sigmoid colon, cecum, and small intestine and, rarely the stomach [1]. Torsion of the gallbladder is a rare clinical condition of the hepatobiliary system, with a reported clinical incidence of 1 in 365,520 hospital admissions. Except for isolated cases reported in children this disease is more frequent in elderly patients with a peak incidence in the 65-75 year-old groups, and a 3:1 female predominance [6]. The anatomical variations of the peritoneal coverings of the gallbladder are well known. There are five recognized positions of the gallbladder in relation to the liver (Carter et al. 1963): (1) completely embedded in the liver; (2) closely attached to the undersurface of the liver by the peritoneum; (3) a complete mesentery but held closely to the liver; (4) a complete mesentery which is long and allows the gallbladder to hang freely; (5) an incomplete mesentery which is attached along the cystic duct and allows the gallbladder to hang freely in the peritoneal cavity. Only situations 4 and 5 can predispose to torsion of the gallbladder [7]. In the elderly, loss of visceral fat with liver atrophy can result in acquired long mesentery. The symptoms of gallbladder torsion are largely non-specific and this makes preoperative diagnosis difficult on the basis of history and physical examinations alone.

Patient typically presents with acute onset abdominal pain with or without vomiting. There may be the presence of a tender mobile mass indicating a 'floating gallbladder [2]. The twist is either clockwise or anti-clockwise. It has been suggested that, if the former, peristalsis of the colon may be responsible; if the latter, then peristalsis of the stomach may initiate the rotation [8]. Specific ultrasound signs seen with gallbladder torsion include the presence of the gallbladder outside its normal anatomic fossa, inferior to the liver or in a transverse orientation with an echogenic conical structure - the twisted pedicle [9]. Computed tomographic scan provides similar diagnostic clues with ultrasonography: the presence of gallbladder outside its fossa and inferior to the liver, pericholecystic fluid, and massively distended gallbladder with wall thickening. Magnetic resonance imaging findings include high signal intensity within the gallbladder wall on T1- weighted images suggesting necrosis and haemorrhage and consistent with gallbladder torsion. The magnetic resonance cholangiopancreatography (MRCP) findings were defined by Usui et al as a v-shaped distortion of extrahepatic bile ducts as a result of traction by the cystic duct, tapering interruption of the cystic duct, a distended gallbladder at the end of the cystic duct which was deviated to the midline, and a difference in intensity between the gallbladder and the extrahepatic bile ducts and cystic duct. Hydroxyiminodiacetic acid (HIDA) scans were reported in one study to form a "bulls-eye" configuration from the accumulation of radioactivity in the gallbladder [10].

## Conclusion

Gallbladder torsion is rare and therefore requires a high index of suspicion for early preoperative diagnosis and prompt surgical intervention. This diagnosis should be considered in the setting of an elderly woman with atypical or non-resolving symptoms and signs of acute cholecystitis in spite of the use of adequate antibiotic therapy. Increasing incidence of gallbladder torsion is being encountered today and this is probably due to unreserved use of imaging investigations and laparoscopy. Early diagnostic imaging investigations and prompt cholecystectomy is the aim in order to achieve best patient outcome. Treatment consists of cholecystectomy with prior detorsion to avoid injury to the common duct. Prognosis is excellent.

## Consent

Written informed consent was obtained from the patient for publication of this case report and accompanying images. A copy of the written consent is available for review by the Editor-in-Chief of this journal.

## Competing interests

The authors declare that they have no competing interests.

## Authors' contributions

VG prepared the case report and reviewed the literature. VS prepared the manuscript and literature search. SV and AS participated in the admission and the care of this patient, the conception, design, data collection and interpretation, analysed the article and made necessary corrections All authors read and approved the final manuscript.

## Supplementary Material

Additional file 1 **Video file showing the cholecystectomy being performed.** Duration 1 min 54 sec, Bit rate 377 kbps, Dimensions 320 × 240, Size 5.19 MB Click here for file
